# Using protease inhibitors to prevent intraperitoneal adhesions: effects of nafamostat mesylate, UAMC-00050, GM6001, and enoxaparin in the cecal ligation and puncture model and the ischemic button model

**DOI:** 10.1007/s00595-025-03166-z

**Published:** 2025-11-14

**Authors:** Philip Plaeke, Joris De Man, Gun-Soo Hong, Michelle de Bruyn, Ingrid De Meester, Philippe G Jorens, Koen Augustyns, Joerg C Kalff, Sven Wehner, Guy Hubens, Benedicte De Winter

**Affiliations:** 1 Laboratory of Experimental Medicine and Pediatrics (LEMP), University of Antwerp, Universiteitsplein 1, Antwerp, 2610 Belgium; 2 Department of Abdominal Surgery, Antwerp University Hospital, Drie Eikenstraat 655, Edegem, 2650 Belgium; 3 Infla-Med, research consortium of excellence, University of Antwerp, Universiteitsplein 1, Antwerp, 2610 Belgium; 4 Department of General, Visceral, Thorax and Vascular Surgery, University Hospital Bonn, Bonn, Germany; 5 Laboratory of Medical Biochemistry, University of Antwerp, Universiteitsplein 1, Antwerp, 2610 Belgium; 6 Department of Intensive Care, Antwerp University Hospital, Drie Eikenstraat 655, Edegem, 2650 Belgium; 7 Laboratory of Medicinal Chemistry, University of Antwerp, Universiteitsplein 1, Antwerp, 2610 Belgium; 8 Antwerp Surgical Training, Anatomy and Research Centre (ASTARC), University of Antwerp, Drie Eikenstraat 655, Edegem, 2650 Belgium; 9 Department of Gastroenterology and Hepatology, Antwerp University Hospital, Drie Eikenstraat 655, Edegem, 2650 Belgium

**Keywords:** Adhesion, Adhesiolysis, Proteases, Protease inhibition, Enoxaparin, Nafamostat, Peritonitis

## Abstract

**Purpose:**

Intraperitoneal adhesions, a major cause of post-surgical intestinal obstruction, arise from an imbalance between proteases of the coagulation and fibrinolysis pathways. This study aimed to reduce early adhesion formation by using the protease inhibitors, nafamostat mesylate (NFM), UAMC-00050, enoxaparin, and GM6001, in the cecal ligation and puncture (CLP) model and the ischemic button (IB) model in mice.

**Methods:**

Mice subjected to CLP received NFM (1, 10, or 20 mg/kg), UAMC-00050 (1 or 5 mg/kg), enoxaparin (1, 5, or 10 mg/kg), or GM6001 (100 mg/kg) in preventive, delayed, and combined setups. Adhesion severity was assessed 48 h post-CLP based on the extent, tenacity, and surgical access time. NFM and enoxaparin were tested further for 7 days in the IB model. Protease activity and gene expression were analyzed in NFM-treated mice.

**Results:**

CLP induced adhesions more strongly than the sham procedure. Preventive NFM reduced the adhesion extent by 49.8%. Repeated enoxaparin administration reduced the extent, tenacity, and access time (-46%). UAMC-00050 and GM6001 had no effect. In the IB model, enoxaparin, but not NFM, reduced the adhesion surface area and tenacity.

**Conclusions:**

Enoxaparin and NFM reduced adhesions effectively, suggesting that coagulation inhibition plays a key role. These findings suggest that selective protease inhibitors, when administered in a timely manner, could reduce intraperitoneal adhesions.

## Introduction

Intraperitoneal adhesions are common after abdominal surgery, occurring after an estimated 67%-100% of laparotomies and after up to 48% of laparoscopic procedures [1, 2]. Although the adhesions are often asymptomatic, they can cause intestinal obstruction, bowel ischemia, and infertility, in up to 20% of patients [1, 3]. Moreover, adhesions complicate abdominal access during subsequent surgical procedures. Intraperitoneal adhesions develop as a result of peritoneal injury or intra-abdominal inflammation, leading to the release of inflammatory mediators and an influx of inflammatory cells [4, 5]. A subsequent imbalance between the coagulation and fibrinolytic system, with stimulation of the coagulation system and inhibition of the fibrinolytic system, has been proposed as a driving factor in the pathophysiology of adhesions [5]. The resulting deposition of fibrin acts as scaffolding for the development of mature fibrous adhesions when extracellular matrix is deposited. The key regulators that have been identified in these pathways are proteases and protease inhibitors, which are involved in the coagulation, fibrinolysis, and inflammatory pathways, and they are activated by intraperitoneal injury or inflammation [5–11].

Based on the central role of proteases in the pathophysiology of intraperitoneal adhesions, this study was conducted to investigate the potential of Nafamostat Mesylate (NFM), UAMC-00050, Enoxaparin, and GM6001 for reducing and preventing the early stages of abdominal adhesions. Recognizing the dual role of proteases in both inflammation and adhesion development, these inhibitors were tested in two animal models: the cecal ligation and puncture (CLP) model, where adhesions are induced because of severe intraperitoneal inflammation, and the ischemic button (IB) model where adhesions develop through localized peritoneal injury.

## Methods

### Study overview

**Fig. 1:**
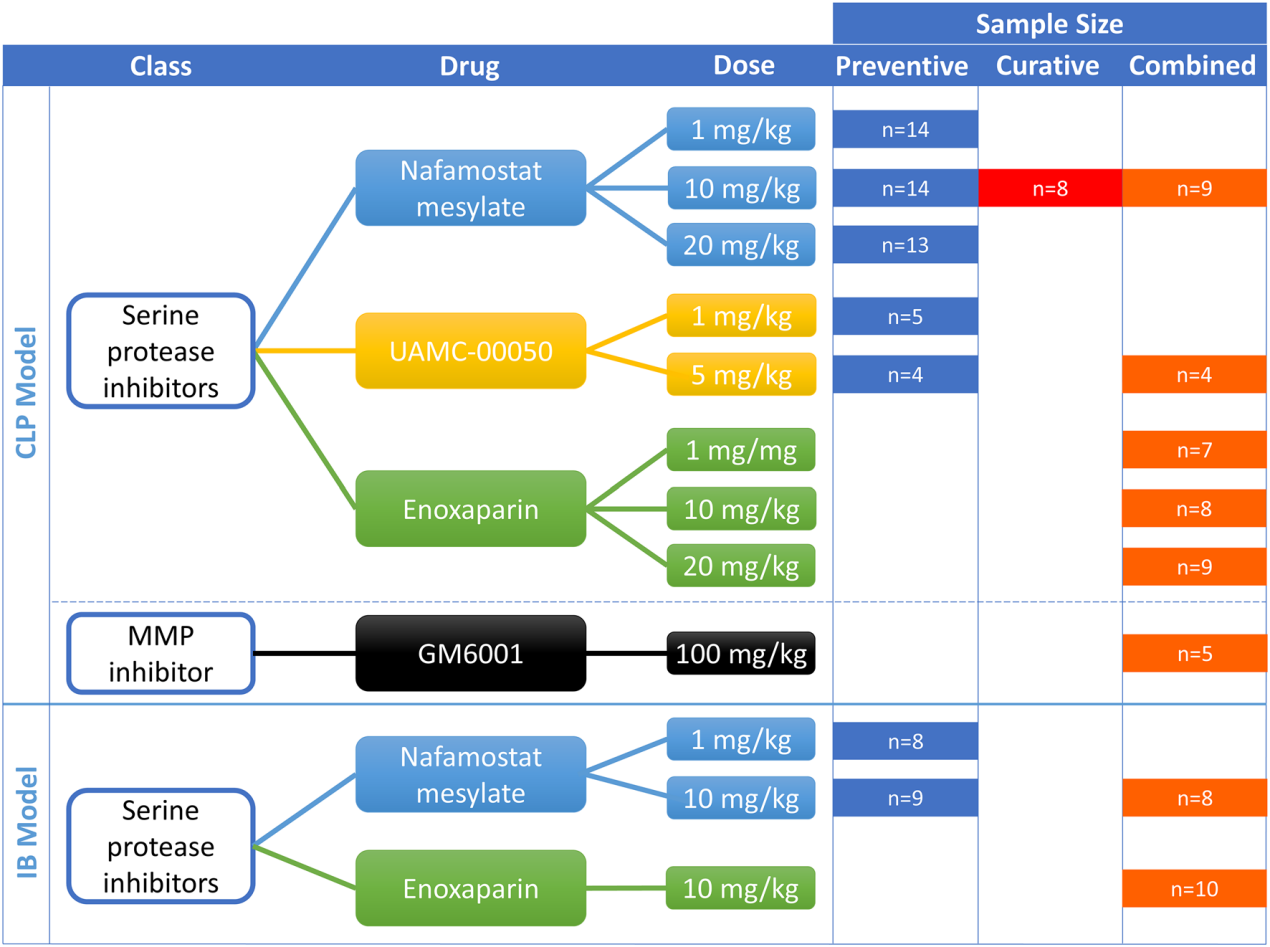
Detailed overview of the protease inhibitors used, the tested doses, and the sample size for each group. Compounds were differentiated according to the class of protease inhibitor. The top part of the figure shows the setup for the CLP model, and the lower part shows the setup for the ischemic button model. CLP, cecal ligation and puncture; IB, ischemic button; MMP, matrix metalloproteinase; n, number of animals per group

We used a staged experimental approach to address the multiple objectives of this study. First, we developed a composite scoring system to standardize the assessment of adhesion formation in the CLP model. Additionally, the baseline level of adhesion formation in the CLP model was assessed, and inflammatory markers in relation to adhesion development were analyzed. In the second stage, the efficacy of NFM, UAMC-00050, enoxaparin, and GM6001 were investigated in the CLP model to assess their effects on intraperitoneal adhesion formation and their ability to modulate intraperitoneal protease activity and intestinal inflammatory pathways. Table 1 and Supplementary Material 1 summarize the details about these protease inhibitors [12–23]. Figure 1 gives the doses for the different experiments. In the third stage, we further evaluated the protease inhibitors that demonstrated anti-adhesion effects in the CLP model and in the ischemic button model.

**Table 1:** Overview of the protease inhibitors and their targets

Protease Inhibitor	Type of inhibitor	Spectrum	Target(s)
Nafamostat Mesylate	Serine protease	Broad	*Coagulation System*: **Thrombin**, **FXa**, FXIa, **FXIIa**, **TF-FVIIa** *Fibrinolysis*: **Plasmin**, PAI 1-3, **uPA**, **tPA** *Complement system*: **C1r**, **C1s**, Factor B, Factor D *Trypsin-like*: **β-tryptase**, **trypsin** *Membrane-anchored*: **TMPRSS2**, **Matriptase**, Hepsin *Kallikreins*: **KLK1**, **KLK2** *Neutrophil proteases*: neutrophil elastase
UAMC-00050	Serine protease	Broad	Coagulation system: Thrombin, FXa Finrinolysis: Plasmin, tPA, uPA Trypsin-like: **β-tryptase** *Membrane-anchored*: **Matriptase** *Kallikreins*: **KLK4**,** KLK8** *Neutrophil proteases*: neutrophil elastase
Enoxaparin	Serine protease	Narrow	*Coagulation system*: **FXa**, Thrombin
GM6001	Matrix Metalloproteinase	Broad	*MMPs*: **MMP-1**, **MMP-2**, **MMP-3**, MMP-7, **MMP-8**, **MMP-9**, MMP-12, MMP-14, MMP-26

Description of the type and targets of the proteases inhibitors used during the experiments. The main targets of the protease inhibitors with high inhibitory potency are shown in bold. F, Factor; PAI, Plasminogen Activator Inhibitor; KLK, Kallikrein; TMPRSS2, Transmembrane protease, serine 2; MMP, Matric Metalloproteinase. Additional details of the protease inhibitors are provided in Supplementary Material 1

### Animals

OF1 mice, aged 8 weeks (Charles River Laboratories, Écully, France), were used for all experiments. To minimize variability introduced by hormonal differences, only male mice were used [24]. Mice were housed in a temperature-controlled (21 °C ± 1 °C) and humidity-controlled (40–60%) environment, with access to standard chow and tap water. Mice were acclimatized for 10 days prior to the experiments. Sample sizes were calculated based on prior experiments (8–10/group) and are presented in Fig. 1 for each arm of the experiment. All experiments were approved by the Animal Ethics Committee of the University of Antwerp (File number ECD2016-68). Experiments were conducted in accordance with the ARRIVE guidelines.

### Procedures

Adhesions were induced using two techniques: the cecal ligation and puncture procedure (CLP) and the ischemic button technique. The CLP procedure is currently the gold standard for inducing and mimicking peritonitis and systemic sepsis, but it also causes significant adhesions through the severe intraperitoneal inflammation. A CLP procedure with 50% cecal ligation and 21G puncture was performed as described previously (Supplementary Material 2) [25–29]. The second procedure, the ischemic button model, creates intraperitoneal adhesions following the creation of four peritoneal buttons. The ischemic button procedure was conducted as described previously (Supplementary Material 2) [30, 31].

### Experimental design

**Fig. 2:**
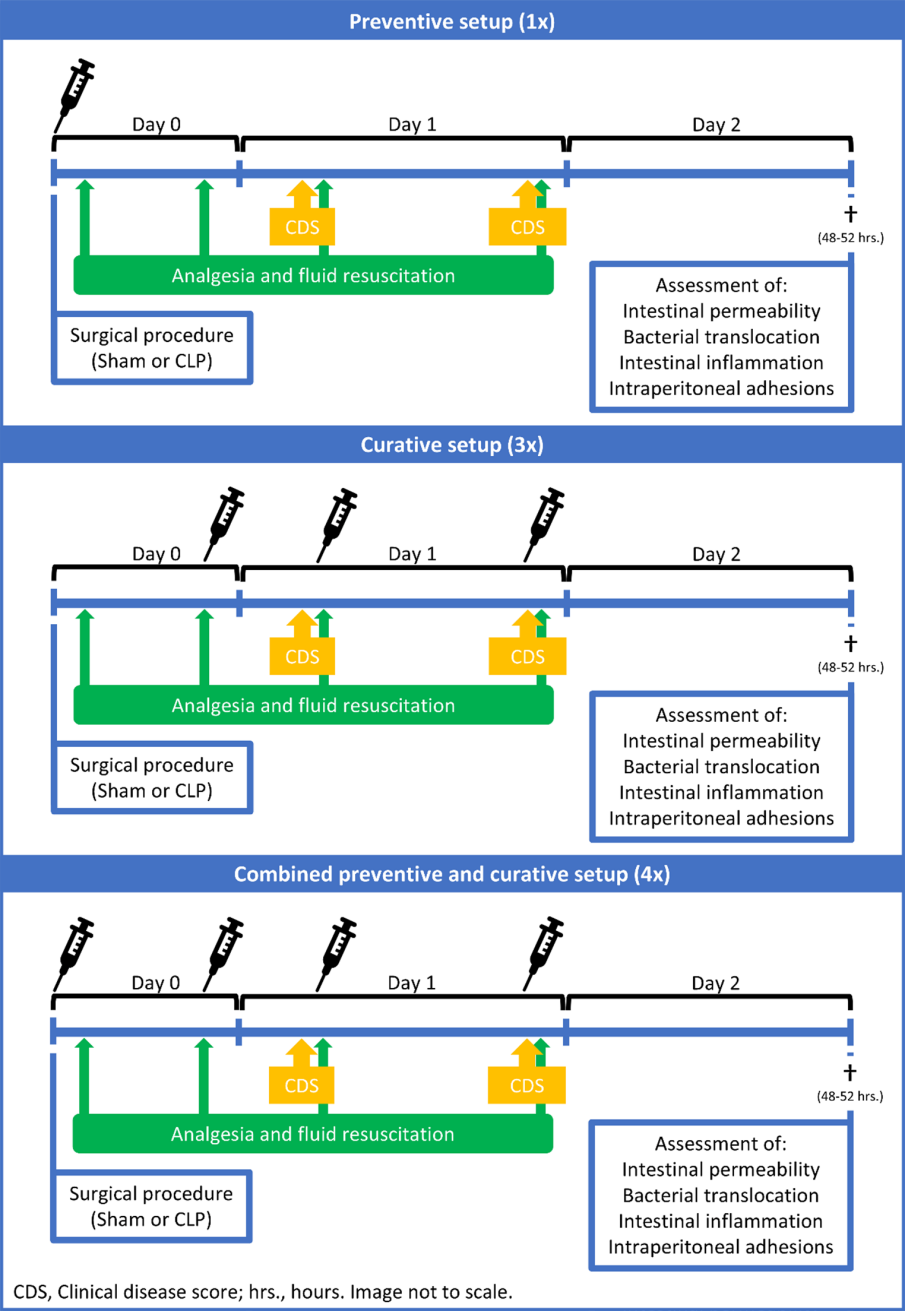
Graphical presentation of the treatment protocols used to investigate the effects of nafamostat mesylate (NFM), UAMC-00050, enoxaparin, and GM6001. Adhesions were induced on day 0 by the CLP procedure, and a sham procedure was performed as a reference. During the next 48 h. mice were either treated in a preventive setup (1x compound administered), curative setup (3x compound administered), or a combined setup (4x compound administered). Vehicles were administered in similar protocols. During the experiment, mice were monitored clinically and received both fluid resuscitation and analgesia. After 48 h, adhesions were scored using an adhesion score. CLP, cecal ligation and puncture; CDS, clinical disease score; hrs., hours

The experimental designs of the CLP and ischemic button models differed. Figure 2 provides an overview of the setups in the CLP model and Fig. 1 gives a graphical overview of the different compounds, treatment setups, and their doses. Doses were calculated based on previous experiments.

In the CLP model, mice were randomized to undergo either the CLP or sham procedure and were also randomly assigned to a treatment group. NFM (1, 10 and 20 mg/kg; Selleckchem, Houston, TX, USA) was administered in three setups: as a single preventive dose, a curative setup (10 mg/kg), or a combined setup (10 mg/kg). The setups were designed to simulate potential clinical applications. In the *preventive regimen*, a single dose was administered intraperitoneally at the start of the CLP procedure, coinciding with the time of peritoneal injury. This setup was intended to demonstrate the usability of the drug to prevent adhesion formation during abdominal surgery. In the second setup, called the *curative setup*, NFM was injected 12 h after the CLP procedure and then given every 12 h three times, until euthanasia. This curative setup was intended to allow us to observe the effects of NFM on adhesions when treatment was delayed. In the third setup, called the *combined setup*, a single preventive dose was followed by three 12-hourly postoperative doses of the curative setup. The animals were euthanized 48 h after the CLP procedure, and the adhesion formation was evaluated immediately.

Based on the results of the NFM experiments, further experiments with UAMC-00050 (1 or 5 mg/kg), GM6001 (100 mg/kg), or enoxaparin (1, 10 or 20 mg/kg; Sanofi, Paris, France) in the CLP model were conducted. Considering the therapeutic half-life and effects in the NFM model, enoxaparin and GM6001 were used only in a combined setup, and thus administered at the start of the CLP procedure and repeated three times every 12 h. UAMC-00050 was given both as a single preventive dose and in a combined setup (5 mg/kg only). UAMC-00050 was dissolved in 5% DMSO. GM6001 (Sigma Aldrich, Saint Louis, MO, USA) was dissolved in 2% DMSO. Since only NFM and enoxaparin affected adhesion formation in the CLP model, only NFM (1 or 10 mg/kg) and enoxaparin (10 mg/kg) were tested in the ischemic button model. For the ischemic button experiments, mice were assigned randomly to different treatment strategies prior to the procedure. Treatments were administered during the first 48 h post-procedure, as either a preventive regimen or a combined setup. Mice were monitored for 7 days, after which they were euthanized and adhesions were evaluated.

For each treatment, an appropriate vehicle control group (saline for enoxaparin, sterile aqua for NFM, and DMSO for GM6001 and UAMC-00050) was included. All drugs were administered intraperitoneally, either directly into the peritoneal cavity under visual control during the procedure or via transcutaneous injection for subsequent administrations.

### Measuring the severity of adhesions

**Fig. 3:**
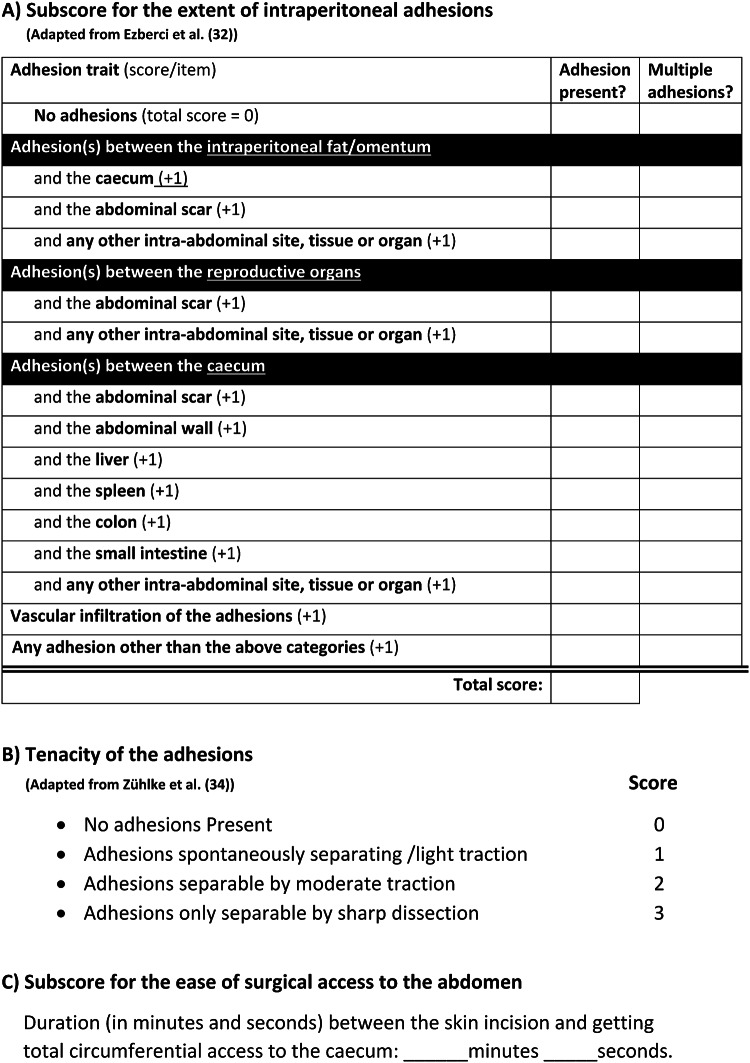
Overview of the composite adhesion score consisting of three individual subscores. The “extent of the adhesions subscore” specifies the number of adhesions and which tissues were involved in the adhesions. One point was added for every characteristic. The total extent score is the sum of all individual scores. The presence of multiple adhesions was registered but does not count toward the total score. The “tenacity of the adhesions subscore” demonstrates the strength and thus the structural organization of the adhesion. For multiple adhesions, the highest score counts. The “subscore for the ease of surgical access to the abdomen” was based on the time in minutes between the start of the midline incision and obtaining full access to the cecum

Intraperitoneal adhesions were evaluated in the CLP and ischemic button models, respectively, 2 days and 7 days post-surgery. To preserve adhesions near the midline incision, a new laparotomy was performed parallel to the previous incision. In the CLP experiments, adhesions were scored using a composite macroscopic adhesion score consisting of three separate subscores (Fig. 3). The first subscore, adapted from Ezberci et al., assessed the extent of adhesions based on the number of tissues and organs involved [32]. Each organ or tissue involved in the adhesions was counted, and the sum represented the total extent of the adhesions. The second subscore, adapted from Zühlke et al., evaluated the tenacity of the adhesions [33, 34]. The third subscore was based on the duration from the start of the abdominal incision to the point at which the terminal ileum was completely accessible, serving as a timed marker for surgical ease of access to the abdomen.

In the ischemic button model, adhesions develop only near the ischemic buttons; therefore, each of the four buttons was scored based on the number of adhesions, their tenacity, and the total surface area involved. The results from all four buttons were subsequently combined to assess the overall treatment effect.

### Quantification of adhesion-related mediators and intraperitoneal protease activity in the CLP model

RT-qPCR was performed as described previously, on full-thickness terminal ileum segments to quantify intraperitoneal inflammation and protease activity in the CLP model and during the NFM experiments [25, 35]. To detect the presence of intraperitoneal proteases and identify potential targets for protease inhibition during CLP-induced inflammation, protease activity was assessed in a small but representative sample (*n* = 5) of sham mice and CLP mice. Proteolytic activities were measured using a Bradford assay, as described previously [17, 36, 37]. Both techniques are explained in detail in Supplementary Material 2 [35].

### Statistical analysis

Clinical outcomes and weight loss were compared statistically across different groups using a student’s t-test for comparisons between two groups, an ANOVA with Tukey HSD post-hoc test for multiple groups, and repeated measures analysis for weight loss analysis over the duration of the experiment, as appropriate. Adhesion scores were analyzed using ANOVA with Dunnett’s post-hoc test, except for the number of adhesions in the IB model where a Chi-Square analysis was used. Protease activity was analyzed using a Mann-Whitney U test. Bivariate correlation analyses were conducted using the Pearson test, with p-values below 0.05 considered significant. Normality of data was assessed using the Kolmogorov-Smirnov test. All p-values were two-sided. All results are presented as means ± standard deviation. Statistical analyses were performed using SPSS version 29 (IBM, Chicago, IL, USA). Results were visualized graphically with GraphPad Prism software, displaying graphs, means, and plotted standard errors of the means (GraphPad Software version 7, La Jolla, CA, USA).

## Results

### Effects of the CLP model on intraperitoneal inflammation and adhesions

To understand the effect of the CLP model on intraperitoneal adhesion development, a sham group of mice receiving a vehicle (*n* = 11, saline 0.9%) was compared with a group of mice that received the vehicle and were subjected to the CLP procedure (*n* = 24). The sham mice did not develop abdominal sepsis, and their fluid and food intake remained stable, with an average weight loss of only 3.72% (SD ± 3.48) after 2 days (Table 2). In contrast, the CLP procedure resulted in an average weight loss of 9.70% (SD ± 2.62, *p* = 0.002) and a significantly higher clinical disease score (*p* < 0.001). No adhesions were observed in the sham group, with the time to achieve complete circumferential access to the cecum averaging 2.46 ± 0.25 min. In contrast, the CLP procedure resulted in significant adhesion formation, with mice scoring an average of 5.97 ± 1.52 (*p* < 0.001) on the extent subscore, 1.67 ± 0.48 (*p* < 0.001) on the tenacity subscore, and an average access time of 6.02 ± 1.80 min (*p* < 0.001).

**Table 2:** Clinical outcomes and weight loss in the different treatment arms of the cecal ligation and puncture (CLP) model

Group (Number of times administered)	CDS ±SD Day 2	Weight loss (%, ±SD)		
		24 h	36 h	48 h
**Sham procedure**				
Vehicle	0.00 ±0.00	2.23 ±2.53	3.48 ±3.12	3.72 ±4.73
**CLP procedure**				
Vehicle	4.79 ±1.14	7.58 ±1.59	8.65 ±2.30	9.70 ±2.62
**CLP + NFM**				
NFM 1 mg/kg (1x)	4.86 ±1.03	6.77 ±2.35	8.17 ±1.94	9.28 ±2.04
NFM 10 mg/kg (1x)	4.36 ±1.01	8.94 ±3.33	9.16 ±2.03	9.96 ±2.54
NFM 20 mg/kg (1x)	4.31 ±0.48	7.41 ±2.40	8.44 ±1.88	9.06 ±2.13
NFM 10 mg/kg (3x)	4.38 ±0.74	8.15 ±1.77	8.54 ±2.01	10.26 ±3.41
NFM 10 mg/kg (4x)	5.00 ±1.22	9.11 ±3.48	10.25 ±3.24	12.94 ±2.68
**CLP + UAMC-00050**				
UAMC 1 mg/kg (1x)	4.20 ±0.84	8.64 ±2.52	7.26 ±5.19	9.44 ±1.98
UAMC 5 mg/kg (1x)	4.00 ±0.82	9.41 ±2.72	11.27 ±0.94	11.50 ±1.23
UAMC 5 mg/kg (4x)	3.75 ±0.50	8.55 ±0.50	10.05 ±2.15	11.97 ±2.53
**CLP + Enoxaparin**				
Enoxaparin 1 mg/kg (4x)	4.43 ±0.53	6.54 ±2.32	8.53 ±2.45	11.23 ±2.95
Enoxaparin 5 mg/kg (4x)	4.38 ±0.74	8.62 ±1.65	8.60 ±1.92	9.73 ±1.73
Enoxaparin 10 mg/kg (4x)	4.78 ±0.97	7.89 ±1.84	8.98 ±1.50	9.21 ±2.23
**CLP + GM6001 MMP inhibitor**				
GM6001 100 mg/kg (1x)	5.80 ±1.10	8.17 ±2.24	8.46 ±1.39	9.50 ±1.94

Overview of the effects of the different treatment groups and administered compounds on clinical disease scores and weight loss. Weight loss data represents the % of the baseline remaining at the indicated time points ±SD. CDS, Clinical Disease Score; CLP, cecal ligation and puncture procedure; SD, Standard Deviation; NFM, Nafamostat Mesylate

**Fig. 4:**
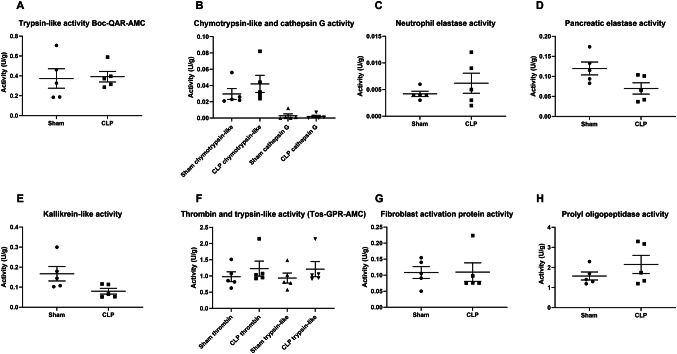
Overview of protease activity in the peritoneal lavage fluid of mice subjected to the sham or CLP procedures (*n*=5/group). Graphs show the measured activities (U/g) for trypsin-like proteases (Boc-QAR-AMC substrate, A), chymotrypsin-like proteases and cathepsin G (B), neutrophil elastase (C), pancreatic elastase (D), kallikrein-like proteases (E), thrombin (F) and trypsin-like (Tos-GPR-AMC substrate, F), fibroblast activation protein (G) and prolyl oligopeptidase (H). The graphs show the mean and standard error of the mean. No significant differences were found between the sham and CLP-mice. An overview of the substrates is provided in Supplementary Material 2

Protease activity, assessed in the peritoneal lavage fluid, was not significantly different between the sham (*n* = 5) and CLP (*n* = 5) groups. The protease activity for thrombin (0.98 ± 0.15 U/g vs. 1.23 ± 0.23 U/g, *p* = 0.347), neutrophil elastase (0.0042 ± 0.0004 U/g vs. 0.0061 ± 0.0019 U/g, *p* = 0.75), and cathepsin G (0.0028 ± 0.0022 U/g vs. 0.0020 ± 0.0013 U/g, *p* = 0.916) was similar for the sham and CLP groups (Fig. 4). Kallikrein-like protease activity seemed to decrease in the CLP mice, but the difference was not statistically different (0.17 ± 0.04 U/g vs. 0.08 ± 0.02, *p* = 0.076). In the CLP model, the gene expression of several mediators could be correlated with the adhesion extent, adhesion tenacity, and abdominal access time (Table 3). More specifically, higher levels of inflammatory cytokines such as interleukin 1 and interleukin 6 correlated with worse intraperitoneal adhesions.

**Table 3:** Correlation between the expression of RT-qPCR in coagulation, inflammation, and adhesion-related mediators and the adhesion subscores

	Adhesion Extent		Adhesion Tenacity		Access Time (min)	
	Pearson *r*	*p*-value	Pearson *r*	*p*-value	Pearson *r*	*p*-value
**Inflammatory Mediators**						
Interleukin 1 (*Il1*)	0.217	0.088	**0.313**	**0.013**	**0.368**	**0.003**
Interleukin 6 (*Il6*)	**0.279**	**0.027**	**0.302**	**0.016**	**0.404**	**0.001**
Interleukin 10 (*Il10*)	0.201	0.114	0.185	0.146	**0.394**	**0.001**
Tumor necrosis factor alpha (*Tnf*)	0.215	0.091	0.177	0.164	**0.422**	**<0.001**
**Coagulation-related Mediators**						
Thrombin (*FII*)	**-0.322**	**0.010**	**-0.366**	**0.003**	**-0.282**	**0.025**
Antithrombin (Serpinc1)	-0.105	0.421	-0.090	0.491	0.001	0.997
Tissue Plasminogen Activator (*Plat*)	0.111	0.386	0.142	0.266	**0.275**	**0.029**
Urokinase Plasminogen Activator (*Plau*)	-0.017	0.895	0.091	0.478	0.113	0.380
Kallikrein-1 (*Klk1*)	**-0.304**	**0.015**	-0.187	0.142	**-0.298**	**0.018**
**Adhesion-related Mediators**						
Matrilysin (*mmp7*)	-0.107	0.405	0.060	0.643	0.014	0.915
Collagenase-2 (*mmp8*)	0.223	0.079	**0.287**	**0.023**	**0.496**	**<0.001**
Gelatinase B (*mmp9*)	0.232	0.067	0.224	0.077	**0.425**	**<0.001**
**Other Mediators**						
Claudin-1 (*Cldn1*)	0.221	0.081	0.174	0.174	**0.391**	**0.002**
Claudin-2 (*Cldn2*)	0.188	0.140	0.241	0.057	**0.403**	**0.001**
Occludin (*Ocln*)	-0.176	0.168	-0.081	0.528	**-0.254**	**0.044**
Tryptase (*Tsab1*)	0.203	0.111	0.168	0.187	**0.380**	**0.002**
Matryptase (*Mtsp1*)	-0.176	0.167	**-0.283**	**0.025**	**-0.469**	**<0.001**
Cathepsine G (*Ctsg*)	-0.032	0.824	-0.124	0.379	0.009	0.950

Correlation between the gene expression of coagulation, inflammation and adhesion-related mediators, and the adhesion subscores. Correlations were calculated using the Pearson statistical test. Significant correlations are presented in bold

### Preventive effects of NFM on intraperitoneal adhesiogenesis in the CLP model

**Fig. 5:**
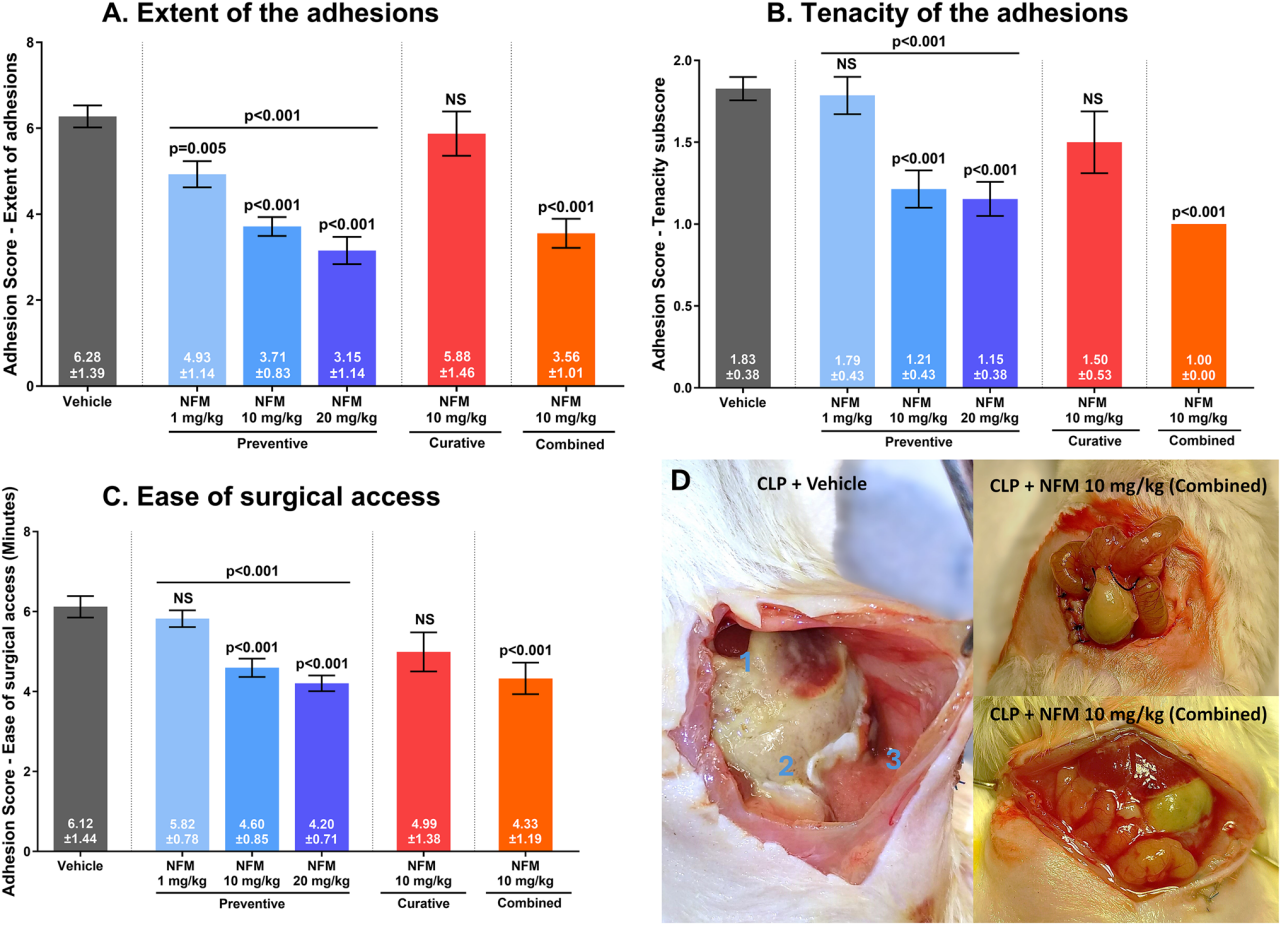
Effects of the preventive, curative, and combined preventive and curative treatment with NFM at doses of 1, 10, and 20 mg/kg (*n*=8-14/group) on the extent of the adhesions (A), tenacity of the adhesions (B), and the ease of surgical access (C). Fig. 5D shows the abdomen 48 h after the CLP procedure in the vehicle-treated group and in the group treated with NFM in a combined 10 mg/kg treatment setup. The abdomen of the CLP+ vehicle group had marked adhesions between the liver [1], omentum with cecum and small bowel [2], and the abdominal wall [3]. In the CLP + NFM 10 mg/kg (combined) group, the ligated cecum had no adhesions or very minor local adhesions. Results are expressed as the mean score ± standard deviation. Error bars display the standard error of the mean. Vehicle-treated CLP mice acted as the reference group. NS, not significant

NFM did not affect the clinical outcomes, compared with the vehicle group (Table 2). The preventive administration of NFM at doses of 1, 10, or 20 mg/kg decreased the extent of the adhesions dose-dependently, with the preventive dose of 20 mg/kg and the combined dose of 10 mg/kg reducing the adhesion extent by 49.8% and 43.3%, respectively (*p* < 0.001, Fig. 5A; Table 4). The curative treatment, where the first dose of NFM was only administered 12 h after CLP, failed to reduce the extent of the adhesions significantly (Fig. 5A). Similarly, NFM, in a preventive or combined treatment regimen, reduced the tenacity of the adhesions and the abdominal access time more significantly than in the vehicle-treated group (Figs. 5B-C; Table 4). Treatment with NFM had no impact on gene expression, except for a significant decrease in the expression of interleukin 1 beta in the 10 mg/kg preventive (-56.08%, *p* = 0.014) and combined (-63.88%, *p* = 0.023) treatment groups (Table 5). Finally, NFM 10 mg/kg was also tested in the sham-operated mice and had no effect on their clinical outcomes. As in the vehicle-treated sham group, none of the sham group mice treated with NFM developed adhesions.

**Table 4:** Effects of Nafamostat Mesylate, UAMC-00050, GM6001, and Enoxaparin on the adhesion scores in the cecal ligation and puncture (CLP) model

	Adhesion Extent		Adhesion Tenacity		Access Time (min)	
	Mean ±SD	*p*-value†	Mean ±SD	*p*-value†	Mean ±SD	*p*-value†
**Nafamostat Mesylate (NFM)**						
- Vehicle	6.28 ±1.39		1.83 ±0.38		6.12 ±1.44	
** Preventive setup**						
- NFM 1 mg/kg	4.93 ±1.14	0.005	1.79 ±0.43	NS	5.82 ±0.78	NS
- NFM 10 mg/kg	3.71 ±0.83	<0.001	1.21 ±0.43	<0.001	4.60 ±0.85	<0.001
- NFM 20 mg/kg	3.15 ±1.14	<0.001	1.15 ±0.38	<0.001	4.20 ±0.71	<0.001
** Curative setup**						
- NFM 10 mg/kg	5.88 ±1.46	NS	1.50 ±0.53	NS	4.99 ±1.38	NS
** Combined setup**						
- NFM 10 mg/kg	3.56 ±1.01	<0.001	1.00 ±0.00	<0.001	4.33 ±1.19	<0.001
**UAMC-00050**						
- Vehicle	4.25 ±0.96		1.25 ±0.50		3.56 ±0.13	
** Preventive setup**						
- UAMC-00050 1 mg/kg	5.00 ±1.41	NS	1.40 ±0.55	NS	4.06 ±0.13	NS
- UAMC-00050 5 mg/kg	4.00 ±0.82	NS	1.00 ±0.00	NS	4.06 ±0.13	NS
** Combined setup**						
- UAMC-00050 5 mg/kg	4.75 ±0.96	NS	1.25 ±0.50	NS	4.94 ±0.97	0.010
**GM6001**						
- Vehicle	5.71 ±1.70		1.71 ±0.49		7.07 ±2.11	
** Combined setup**						
- GM6001 100 mg/kg	4.60 ±2.79	NS	1.60 ±0.89	NS	6.50 ±2.50	NS
**Enoxaparin**						
- Vehicle	6.25 ±1.58		1.88 ±0.13		4.96 ±0.98	
** Combined setup**						
- Enoxaparin 1 mg/kg	5.71 ±0.95	NS	1.57 ±0.20	NS	3.93 ±0.75	0.05
- Enoxaparin 5 mg/kg	4.13 ±0.64	0.001	1.50 ±0.19	NS	3.47 ±0.41	<0.001
- Enoxaparin 10 mg/kg	2.44 ±0.53	<0.001	1.00 ±0.00	<0.001	2.69 ±0.26	<0.001

Overview of the effects of Nafamostat Mesylaten (NFM), UAMC-00050, GM6001 and Enoxaparin on the adhesion extent subscore, adhesion tenacity subscore, and abdominal access time. Results are presented as the mean ±standard deviation (SD). †p-values were calculated using a one-way ANOVA with Dunnett’s post-hoc test, comparing the score to the vehicle group for each different protease inhibitor

**Table 5:** Effects of Nafamostat mesylate (NFM) on gene expression in the cecal ligation and puncture (CLP) model

		Nafamostat Mesylate (NFM)			
		Preventive			Combined
	Vehicle	1 mg/kg	10 mg/kg	20 mg/kg	10 mg/kg
**Inflammatory Mediators**					
Interleukin 1 (*Il1*)	1.00 ±0.73	0.98 ±0.57	**0.44 ±0.26†**	0.55 ±0.17	**0.36 ±0.26†**
Interleukin 6 (*Il6*)	1.00 ±0.48	0.78 ±0.33	0.66 ±0.52	**0.44 ±0.28‡**	0.57 ±0.40
Interleukin 10 (*Il10*)	1.00 ±0.72	1.84 ±1.94	1.09 ±0.82	0.63 ±0.48	0.59 ±0.43
Tumor Necrosis Factor Alpha (*Tnf*)	1.00 ±0.45	1.22 ±0.72	0.86 ±0.44	0.67 ±0.27	0.72 ±0.30
**Coagulation-related Mediators**					
Thrombin (*FII*)	1.00 ±0.61	0.86 ±0.31	1.30 ±0.46	1.09 ±0.21	1.31 ±0.30
Antithrombin (*Serpinc1*)	1.00 ±0.89	0.65 ±0.25	1.55 ±2.24	1.45 ±1.42	1.58 ±0.68
Tissue Plasminogen Activator (*Plat*)	1.00 ±0.55	1.54 ±0.97	1.09 ±0.48	1.14 ±0.39	1.37 ±0.61
Urokinase Plasminogen Activator (*Plau*)	1.00 ±0.39	1.14 ±0.58	1.15 ±0.51	1.13 ±0.40	1.12 ±0.45
Kallikrein-1 (*Klk1*)	1.00 ±0.42	0.98 ±0.46	1.25 ±0.39	1.31 ±0.39	1.20 ±0.35
**Adhesion-related Mediators**					
Matrilysin (*mmp7*)	1.00 ±0.50	1.01 ±0.40	0.98 ±0.36	1.10 ±0.39	0.79 ±0.24
Collagenase-2 (*mmp8*)	1.00 ±0.61	1.59 ±1.11	0.73 ±0.46	0.69 ±0.44	0.56 ±0.31
Gelatinase B (*mmp9*)	1.00 ±0.45	1.28 ±0.79	0.79 ±0.36	0.75 ±0.36	0.80 ±0.35
**Other Mediators**					
Claudin-1 (*Cldn1*)	1.00 ±0.76	1.60 ±1.96	1.04 ±0.74	0.57 ±0.42	0.62 ±0.46
Claudin-2 (*Cldn2*)	1.00 ±0.34	1.07 ±0.27	0.75 ±0.26	0.79 ±0.31	0.77 ±0.22
Occludin (*Ocln*)	1.00 ±0.45	0.70 ±0.35	0.97 ±0.42	1.17 ±0.31	0.95 ±0.36
Tryptase (*Tsab1*)	1.00 ±0.82	1.73 ±2.00	0.98 ±0.69	0.60 ±0.41	0.42 ±0.29
Matryptase (*Mtsp1*)	1.00 ±0.55	0.71 ±0.33	1.15 ±0.63	1.00 ±0.25	1.58 ±0.86
Cathepsine G (*Ctsg*)	1.00 ±0.38	1.22 ±0.41	1.07 ±0.37	1.47 ± 0.94	1.60 ±1.03

Overview of the gene expression of inflammatory, coagulation, and adhesion-related mediators in the CLP model following NFM administration. Results were compared statistically to the vehicle group using a one-way ANOVA with Dunnett’s post-hoc test. †Indicates p-value <0.05. ‡indicates p-value <0.01

### Effects of UAMC-00050 on intraperitoneal adhesiogenesis in the CLP model

**Fig. 6:**
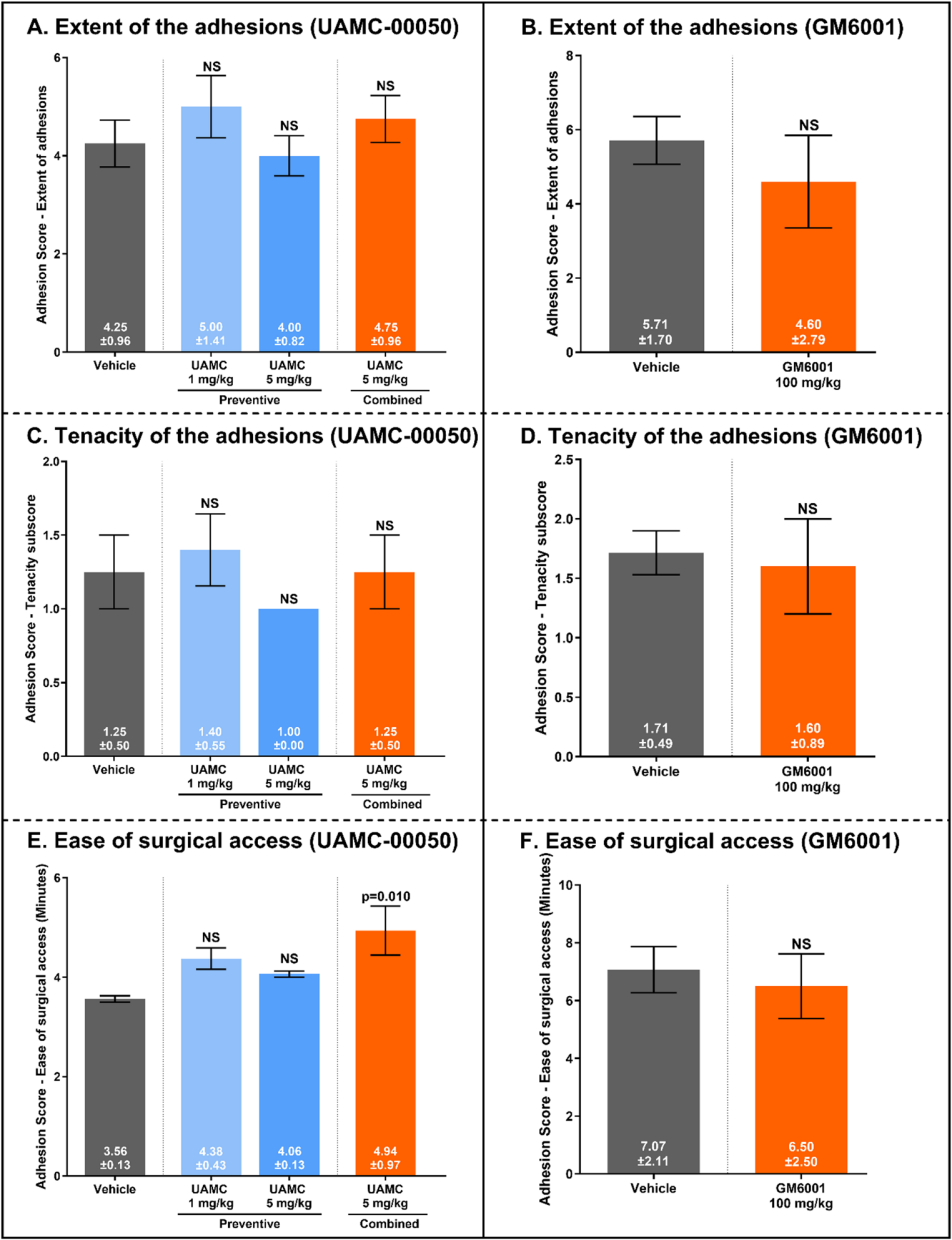
Adhesion scores following the administration of UAMC-00050 and GM6001. No significant effects were found on the extent of the adhesions (6 A and 6B) or on the tenacity of the adhesions (6 C and 6D). UAMC-00050 or GM6001 did not improve the abdominal access time (in minutes). In fact, UAMC-00050 increased the access time significantly, by 38.7%. Results are expressed as the mean score ± standard deviation. Error bars display the standard error of the mean. Vehicle-treated (Saline 0.9% + 5% DMSO) CLP mice (*n*=4) acted as the reference. NS, not significant

UAMC-00050 had no effect on clinical disease scores or weight loss (Table 2). Moreover, none of the dosages or treatment protocols of UAMC-00050 led to significant changes in the extent or tenacity of the adhesions (Table 4; Fig. 6A and C). The only significant effect of UAMC-00050 was in the time it required to gain full access to the cecum (Fig. 6E). However, this effect was negative, increasing the duration by 27.9% (*p* = 0.010), and was observed only in the combined treatment group.

### Effects of GM6001 on intraperitoneal adhesiogenesis in the CLP model

GM6001, a broad-spectrum MMP-inhibitor was administered in a combined setup in mice with CLP-induced sepsis. No clinical effects were observed (Table 2). GM6001 failed to reduce any of the adhesion subscores 2 days after the CLP procedure (Table 4; Fig. 6).

### Effects of Enoxaparin on intraperitoneal adhesiogenesis in the CLP model

**Fig. 7:**
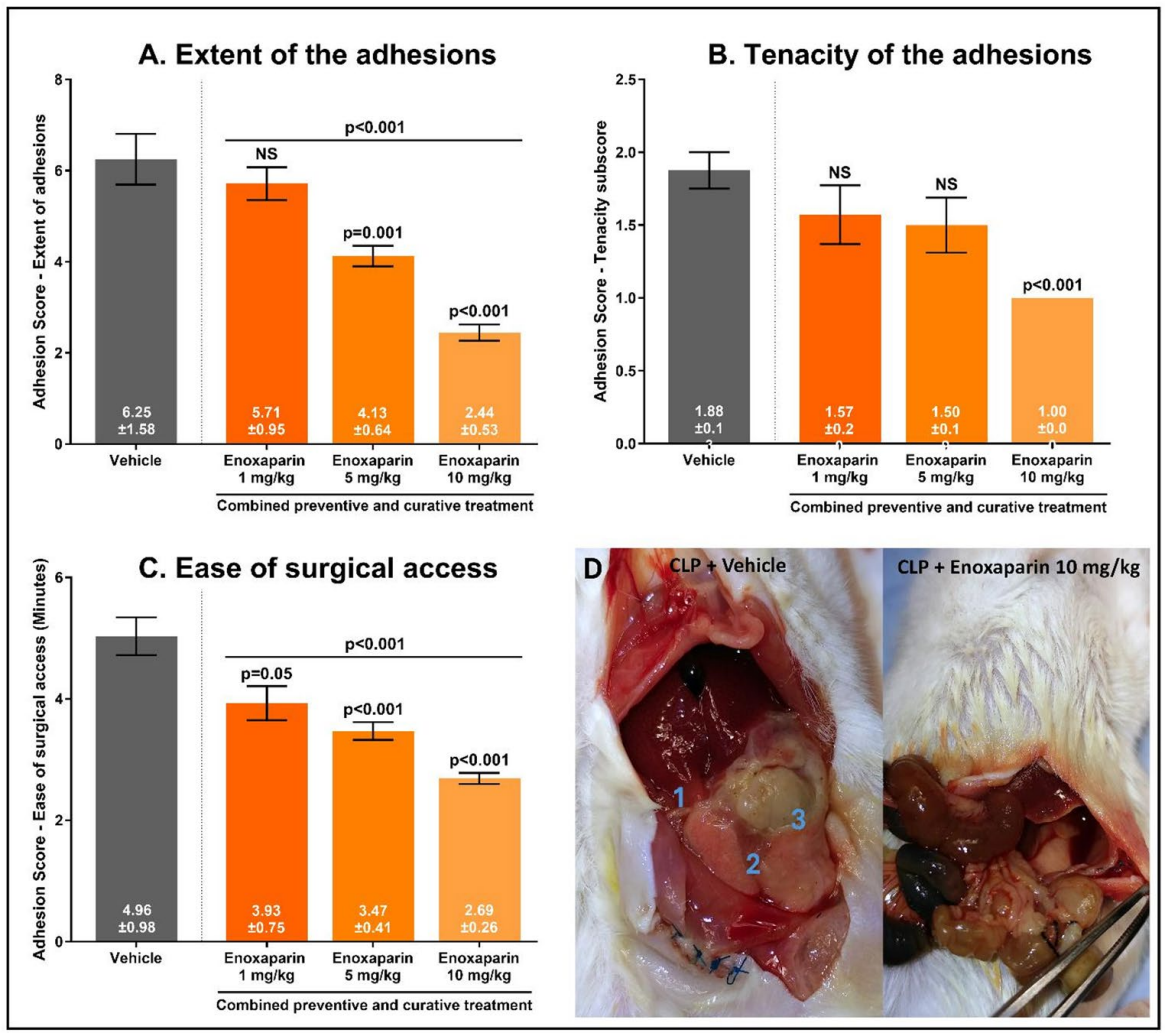
Effects of a combined preventive and curative treatment with enoxaparin given at doses of 1, 5, and 10 mg/kg (*n*=7-9/group) on the extent of the adhesions (A), tenacity of the adhesions (B), and the ease of surgical access (C). Fig. 7D shows the abdomen 48 h after the CLP procedure following vehicle treatment and treatment with enoxaparin 10 mg/kg. In the CLP + vehicle group, the liver [1], cecum [3], omentum and small bowels [2] were affected by adhesions (among other organs). The abdomen of the CLP + enoxaparin 10 mg/kg group was reopened without any major adhesions, and the ligated cecum was free in the abdomen. Results are expressed as the mean score ± standard deviation. Error bars display the standard error of the mean. Vehicle-treated (saline 0.9%) CLP mice (*n*=8) acted as the reference group. NS, not significant

No clinical effects were observed following the administration of enoxaparin (Table 2). Treatment with enoxaparin had a significantly beneficial effect on all the adhesion scores after the CLP procedure (Table 4; Fig. 7). Enoxaparin caused a dose-dependent reduction in the extent of the adhesions, with no effect observed when enoxaparin was administered at 1 mg/kg, but the score decreased by 71% when it was administered at 10 mg/kg (*p* < 0.001, Fig. 7A). Enoxaparin decreased the tenacity of the adhesions by 46.7% (*p* < 0.001) 2 days after the CLP procedure, but only when it was administered at a dose of 10 mg/kg (Fig. 7B). A comparable dose-dependent effect of enoxaparin was observed on the subscore that measured the ease of surgical access and this effect was also seen at the lowest dose of 1 mg/kg (*p* = 0.005, Fig. 7C). An even larger reduction was achieved in the enoxaparin 5 mg/kg (-30.0%, *p* < 0.001) and 10 mg/kg groups (-45.8%, *p* < 0.001). The latter dose facilitated access to the abdomen to such an extent that the duration did not differ from that in the mice subjected to the sham procedure.

### Effects of the protease inhibitors NFM and Enoxaparin in the IB model

**Fig. 8:**
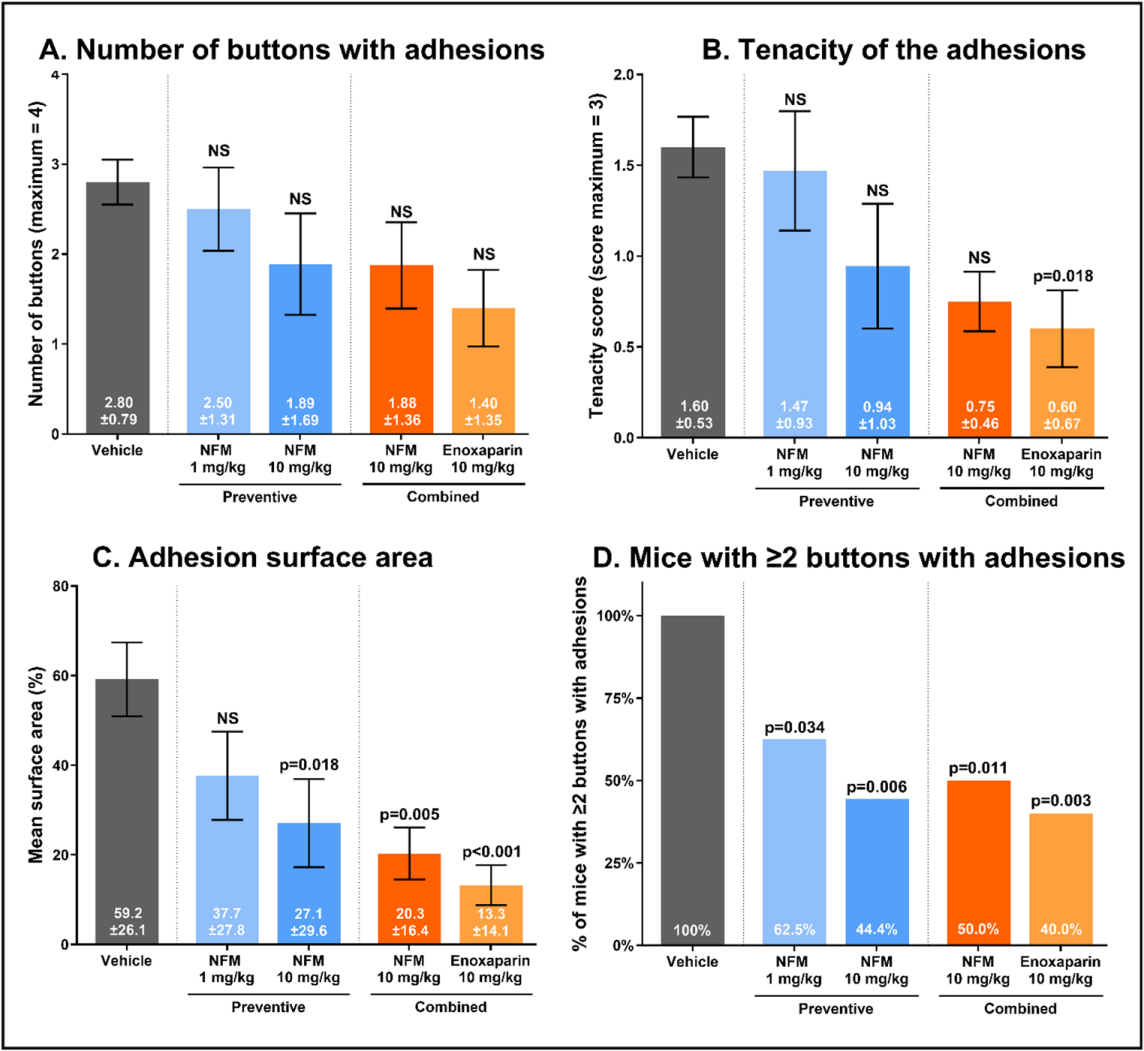
Effects of preventive or combined preventive and curative treatment with NFM (1 mg/kg and 10 mg/kg, *n*=8-10/group) and enoxaparin (10 mg/kg, *n*=10/group) on the number of buttons with adhesions (A), tenacity of the adhesions (B) and surface area (C) in the ischemic button model. Fig. 8D shows the percentage of mice with two or more positive buttons. Results are expressed as the mean score ±standard deviation. Error bars display the standard error of the mean. Vehicle-treated (sterile aqua) CLP mice (*n*=10) acted as the reference group. NS, not significant

During the IB experiments, mice were monitored for 7 days, after which a new laparotomy was performed to score the adhesions. The IB model had minimal clinical impact, with clinical disease scores remaining at zero throughout the experiment, with an average weight loss of 3.31% ± 4.65 from the baseline. NFM and enoxaparin had no effect on weight loss or on the clinical disease score (Table 6). The vehicle-treated mice all developed at least one adhesion, with an average of 2.80 ± 0.79 of 4 buttons with an adhesion, an average tenacity score of 1.60 ± 0.53, and adhesion surface area of 59.2% ±26.1% (Fig. 8). Both NFM and enoxaparin appeared to reduce the number of adhesions in the IB model, in a dose-dependent manner. The combined preventive and curative treatment with NFM 10 mg/kg and enoxaparin 10 mg/kg reduced the number of buttons with adhesions by 33.9% (*p* = 0.09) and 50.0%, respectively (*p* = 0.01). Treatment with both drugs significantly reduced the likelihood of having more than 1 of 4 buttons with an adhesion. Two or more adhesions developed in all of the vehicle-treated IB group mice, but in only 50% (*p* = 0.011) of the NFM-treated mice and 40% (*p* = 0.003) of the enoxaparin-treated mice (Fig. 8D).

**Table 6:** Clinical outcomes and weight loss in the different treatment arms of the ischemic button model

Group (Number of times administered)	CDS ±SD Day 1	Weight loss (%, ±SD)		
		Day 1	Day 2	Day 7
IB procedure				
Vehicle	0.00 ±0.00	8.11 ±5.63	6.42 ±3.38	3.31 ±4.65
**IB + NFM**				
NFM 1 mg/kg (1x)	0.00 ±0.00	4.68 ±3.61	7.39 ±3.73	3.65 ±4.98
NFM 10 mg/kg (1x)	0.00 ±0.00	6.11 ±2.59	7.29 ±2.98	2.17 ±5.09
NFM 10 mg/kg (4x)	0.00 ±0.00	5.43 ±3.71	6.94 ±4.92	2.20 ±4.17
**IB + Enoxaparin**				
Enoxaparin 10 mg/kg (4x)	0.00 ±0.00	6.85 ±4.47	6.77 ±3.11	0.99 ±2.74

Overview of the effects of different treatment groups and administered compounds on clinical disease scores and weight loss. Weight loss data represents the % of baseline remaining at the indicated time points ±SD. CDS, Clinical Disease Score; CLP, cecal ligation and puncture procedure; SD, Standard Deviation; NFM, Nafamostat Mesylate

Enoxaparin 10 mg/kg also reduced the strength of the adhesions, by 67.5% (*p* = 0.018, Fig. 8B). Finally, NFM 10 mg/kg and enoxaparin 10 mg/kg reduced the surface area of the adhesions significantly. For NFM, this effect was observed with a single preventive dose of 10 mg/kg, leading to a 54.2% (*p* = 0.018) lower adhesion surface area. This effect was enhanced further if NFM was administered in a combined 10 mg/kg regimen (-65.1%, *p* = 0.005). The preventive effect of enoxaparin on adhesion formation was observed to be even stronger with a reduction in surface area by 77.5% (*p* < 0.001).

## Discussion and conclusion

Intraperitoneal adhesions are a pathophysiological response of the peritoneum to injury caused by surgery, infection, or inflammation. These adhesions are a major cause of complications, including intestinal obstruction, bowel ischemia, and infertility, often necessitating surgical intervention, which can result in further adhesions. While the mechanisms of adhesion formation are not fully understood, inflammatory, coagulation, and fibrinolytic pathways seem central to this process, with proteases playing key roles.

To investigate this, we administered protease inhibitors in two murine models. The cecal ligation and puncture (CLP) model, the gold standard for sepsis research, was our primary platform because of its reliable adhesion formation and involvement of multiple pathways. However, because the CLP model is complex and relies on inflammation as the driving force for adhesion formation, we added the ischemic button model to validate our findings. The ischemic button model is known to induce adhesions in a more controlled way through peritoneal injury instead of severe intraperitoneal inflammation. Based on current knowledge about adhesiogenesis, we hypothesized that serine proteases such as thrombin, tPA, uPA, and plasmin, as well as matrix metalloproteinases, would be the main targets; and therefore, selected four protease inhibitors to modulate adhesion formation [5–11, 38, 39].

Most intraperitoneal adhesions develop following peritoneal injury, where fibrin scaffolding and centripetal growth of the peritoneum eventually lead to strong fibrous bands. This repair process relies heavily on inflammatory and coagulation cascades [5]. Peritonitis acts as a strong trigger for the development of extensive adhesions since it amplifies these proinflammatory and procoagulant responses. Our findings showed that the CLP model leads to severe intra-abdominal adhesions, making it a suitable screening model for the modulation of intraperitoneal adhesion formation. Treatment with the broad-spectrum serine protease inhibitor NFM reduced intraperitoneal adhesions significantly when given at a minimum dose of 10 mg/kg, but only if administered at least once during the CLP procedure itself: during the preventive and combined setup, not during the curative setup. The efficacy of NFM is likely attributed to its inhibition of the multiple proteases involved in both the coagulation (activated factor Xa, thrombin) and fibrinolysis (tPA, uPA, kallikrein-1, kallikrein-2) pathways [40, 41]. Its known anti-inflammatory effects, such as the inhibition of neutrophil elastase, may also contribute to reducing adhesion formation. This multi-pathway inhibition, which is especially useful in sepsis with its dysregulated proinflammatory state, likely plays a critical role in reducing adhesiogenesis.

Based on these results, we blocked the coagulation pathway more selectively by using enoxaparin, a factor Xa inhibitor. Enoxaparin also reduced adhesion scores significantly across all three subscores, provided it was administered at a sufficient dose (10 mg/kg) in a combined setup. In accordance with our results using NFM, enoxaparin showed a dose-dependent reduction in adhesions, with maximal effects observed at higher doses. No adverse impact of NFM or enoxaparin on clinical outcomes were observed in our murine sepsis model. Adhesion prevention was successful only if NFM or enoxaparin were administered during the procedure. Delayed administration of NFM 12 h post-CLP without a perioperative dose, as seen in the curative setup, failed to reduce adhesions. These findings align with existing literature suggesting a critical intervention window after peritoneal injury [42]. Moreover, this observation highlights that pharmacological strategies should be administered ideally before, during, or immediately following surgery, since the early fibrin deposition seems to mark a “point of no return” beyond which protease inhibitors are less effective [4].

The limited efficacy of UAMC-00050, which weakly inhibits factor Xa (IC_50_ 100 µM), tPA (IC_50_ 7 µM), plasma kallikreins (IC_50_ > 2.5 µM), plasmin (IC_50_ 0.9 µM), and thrombin (IC_50_ 0.39 µM) suggests that inhibition across the coagulation and fibrinolysis pathways is necessary [17, 43]. The ineffectiveness of GM6001 in reducing adhesions is most likely due to the delayed role of MMPs in extracellular matrix remodeling, implying a potential therapeutic role at later adhesion maturation stages rather than early formation. To explore these findings further, we tested NFM and enoxaparin in the ischemic button model, where the peritoneal injury is localized and the inflammation is less extensive. Although NFM appeared to reduce the adhesion severity in the IB model, it proved to be less effective than in the CLP model and did not reach significance. Conversely, enoxaparin retained its beneficial effects on adhesion formation. This suggests that NFM’s efficacy in the CLP model derives from its dual action on inflammation and coagulation. Without strong inflammation, its impact on adhesions diminishes, whereas enoxaparin, which blocks the final step in the coagulation pathway, remains effective. Possibly, the greater variability observed in the IB model, probably because of its extended 7-day course and lower inflammatory intensity, could have accounted for why some of our results did not reach significance.

To date, few studies have explored the pharmacologic approaches to prevent intraperitoneal adhesions [44]. Since adhesions form at sites of peritoneal injury, most research has focused on covering damaged areas with adhesion barriers made from natural polymers such as hyaluronic acid, oxidized and carboxymethyl cellulose, dextran, or chitosan; or synthetic polymers. Although adhesion barriers such as membranes or instilled icodextrin have been advocated in guidelines and marketed for the management of adhesive small-bowel obstruction, their adoption has been limited by challenges like interference with normal wound healing and effectiveness that varies with injury conditions [44, 45]. Since these barriers do not modulate the inflammatory, coagulation and fibrinolytic pathways specific to adhesiogenesis, they are presumed to be less favorable in case of extensive peritoneal injury and intraperitoneal inflammation, such as in our CLP model. Pharmacological prevention using protease inhibitors that directly interfere with these adhesion pathways is expected to be more beneficial for preventing adhesions.

Several studies have investigated the use of proteases and protease inhibitors to prevent the development of intraperitoneal adhesions in animal models [46–65]. Most of these experiments have been executed with tPA, which was able to reduce adhesions significantly by over 50% in various animal models. In contrast, thrombin, plasmin and fibrinolysin proved ineffective for preventing adhesions. Protease inhibitors have been studied less, but aprotinin and chymase inhibitors also appear to reduce the formation of adhesions. GM6001 was used only in one study and also failed to reduce the formation of adhesions [39]. Our results are in line with these experiments, where interference with the coagulation pathway by NFM and enoxaparin, creating a net fibrinolytic effect, seems to reduce adhesions dramatically.

Despite these promising results, several limitations must be considered before clinical translation. First, our findings are based on a sepsis-driven model (CLP), which features sustained peritoneal inflammation driving adhesion formation and it may not yield similar results for minimal or localized peritoneal injury. Thus, to explore more localized peritoneal injury, we also used the ischemic button model, where NFM demonstrated less efficacy, supporting the hypothesis that its anti-adhesive effects may largely derive from its anti-inflammatory properties. This implies that a “one drug fits all” solution for adhesions is unlikely to be possible and that the type of protease inhibitor should be chosen based on the underlying pathology and the severity of inflammation. Moreover, the duration of the procedure might interfere with the processes involved in adhesion formation and since the mice can be observed only for a maximum of 48 h after the CLP procedure due to ethical considerations, we included the ischemic button model, which also suggests a positive effect of protease inhibition. Second, both NFM and enoxaparin inhibit coagulation. While this appears to be central to adhesion formation, full coagulation inhibition immediately post-surgery may increase bleeding risks and impair wound healing. Although no adverse effects were observed in our models, our findings should be viewed as a foundational step with future studies identifying and refining protease targets to balance efficacy and safety. Lastly, our gene expression sampling focused on the terminal ileum to assess inflammation. Although the most severe adhesions were concentrated primarily near the cecum, inflammation and adhesion heterogeneity across the peritoneal cavity could mean that gene expression in the ileum may not fully represent the dynamic processes occurring in the adhesions or directly on the peritoneal surface.

In conclusion, our study findings contribute to the field of adhesion prevention by demonstrating that a pharmacologic intervention with protease inhibitors can reduce adhesion formation effectively in a severe intraperitoneal inflammation model and even showing the dose-dependent effects of NFM and enoxaparin. Furthermore, we confirmed some efficacy of these treatments in the ischemic button model, where inflammation is less intense, thus supporting the versatility of protease inhibitors across different adhesion contexts. From a translational perspective, we demonstrated that prophylaxis for intraperitoneal adhesions can be offered directly during abdominal surgical procedures without the need for additional treatment afterwards. Further research targeting specific proteases involved in adhesion formation is essential to enhance the efficacy of these inhibitors while minimizing side effects. Future studies should assess protease inhibitors across different animal models, and with prolonged follow-up, as adhesion development mechanisms can differ widely, impacting the effectiveness of an inhibitor to reduce adhesions.

## Data Availability

The data that support the findings of this study are available from the corresponding author upon reasonable request. Some data may not be made available because of privacy or ethical restrictions.
